# Coronary Embolism: A Rare Complication After Takotsubo Cardiomyopathy

**DOI:** 10.7759/cureus.113795

**Published:** 2026-08-01

**Authors:** Boran Mao, Sajjal Mahmood, Rohaid Ali, Sankalp Goberdhan, Mohammed Ansari, Sayed T Hussain

**Affiliations:** 1 Internal Medicine, University of Central Florida College of Medicine, Orlando, USA; 2 Internal Medicine, Sheikh Zayed Medical College (SZMC), Rahim Yar Khan, PAK; 3 Internal Medicine, Aga Khan University, Karachi, PAK; 4 Department of Medicine, University of Central Florida - HCA Florida Healthcare, Kissimmee, USA; 5 Cardiology, University of Central Florida College of Medicine, Orlando, USA

**Keywords:** anticoagulation, coronary embolism, hypercoagulability, perioperative management optimization, takotsubo cardiomyopathy

## Abstract

Takotsubo cardiomyopathy (TTC) is a transient stress-induced cardiomyopathy characterized by reversible left ventricular (LV) systolic dysfunction that mimics acute coronary syndrome. Although most patients recover ventricular function, severe LV dysfunction may result in LV thrombus formation and systemic embolization. Coronary embolism is an exceptionally rare complication of TTC and may present as ST-elevation myocardial infarction (STEMI). We report a rare case of coronary embolism complicating TTC in a patient with multiple thromboembolic risk factors. A 74-year-old woman with chronic obstructive pulmonary disease, tobacco use, dyslipidemia, generalized anxiety disorder, and a history of unprovoked deep vein thrombosis and pulmonary embolism on apixaban presented with chest pain and dyspnea two days after cataract surgery. Apixaban had been withheld for three days preoperatively. Electrocardiography demonstrated inferior and anterolateral ST-segment elevation, and cardiac biomarkers were markedly elevated. Emergent coronary angiography revealed embolic occlusion of the distal obtuse marginal branch without obstructive coronary artery disease, requiring thrombectomy and balloon angioplasty. Left ventriculography demonstrated classic apical ballooning with an LV mural thrombus, while transthoracic echocardiography showed an ejection fraction of 15-20% with severe global wall-motion abnormalities. The patient was diagnosed with TTC complicated by LV thrombus and coronary embolism and was treated with dual antiplatelet therapy, anticoagulation, guideline-directed medical therapy, and a wearable cardioverter-defibrillator. This case illustrates the diagnostic challenge posed by the overlapping presentation of TTC and coronary embolism. Severe LV dysfunction, apical akinesis, perioperative interruption of anticoagulation, prior venous thromboembolism, tobacco use, and a newly identified pulmonary nodule likely contributed to a multifactorial hypercoagulable state. Unlike the typical mechanism in which embolization occurs during recovery of ventricular function, coronary embolism developed during the acute phase of TTC. This case also highlights the importance of evidence-based perioperative management of direct oral anticoagulants to minimize thromboembolic risk in high-risk patients. Coronary embolism is a rare but potentially fatal complication of TTC. Early recognition, prompt coronary intervention, anticoagulation, and multidisciplinary management are essential to optimize outcomes. Careful perioperative anticoagulation planning and further research are needed to better identify patients at highest risk for thromboembolic complications and to refine preventive strategies.

## Introduction

Apical ballooning syndrome or Takotsubo cardiomyopathy (TTC) is characterized by transient left ventricular (LV) systolic dysfunction presenting with a clinical picture similar to acute coronary syndrome (ACS) or myocardial infarction (MI). The pathophysiology of TTC involves sudden release of dopamine, epinephrine, and norepinephrine in response to emotional or physical stress that leads to direct myocardial toxicity, microvascular dysfunction, and transient myocardial stunning, causing acute cardiac remodeling affecting both perfusion and cardiomyocyte structure. TTC is primarily diagnosed by using modified Mayo criteria [1]. It includes 1) transient hypokinesis, akinesis, or dyskinesis of the LV mid-segments, with or without apical involvement, such as ballooning; 2) regional wall-motion abnormalities extending beyond a single epicardial vascular distribution; 3) absence of obstructive coronary disease or angiographic evidence of acute plaque rupture; 4) new electrocardiographic abnormalities (either ST-segment elevation and/or T-wave inversion) or modest elevation in cardiac troponin; and 5) absence of pheochromocytoma and myocarditis [1]. Complications include acute heart failure, cardiogenic shock, arrhythmias, LV outflow tract obstruction, LV thrombus formation, and, less commonly, coronary embolism.

Coronary embolism is a rare, nonatherosclerotic cause of acute MI or ACS, accounting for approximately 2-5% of MI cases [2]. The pathophysiology of this complication is that the acute, severe LV dysfunction predisposes to intraventricular thrombus formation, particularly in patients with markedly reduced LV ejection fraction and apical akinesis according to Virchow’s triad. Due to its transient nature, returning to baseline mobility of the ventricles leads to thrombus dislodgement and embolization, causing cerebral, peripheral, or rarely coronary artery ischemia or infarction. It is managed similarly to ACS per the American College of Cardiology (ACC)/American Heart Association (AHA)/American College of Emergency Physicians (ACEP)/National Association of EMS Physicians (NAEMSP)/Society for Cardiovascular Angiography and Interventions (SCAI) Guideline [3]. Common causes include atrial fibrillation, prosthetic heart valves, infective endocarditis, intracardiac tumors, cardiomyopathies, and hypercoagulable states. This case highlights coronary embolism as a rare complication of TTC with multiple contributing factors.

## Case presentation

**Figure 1:**
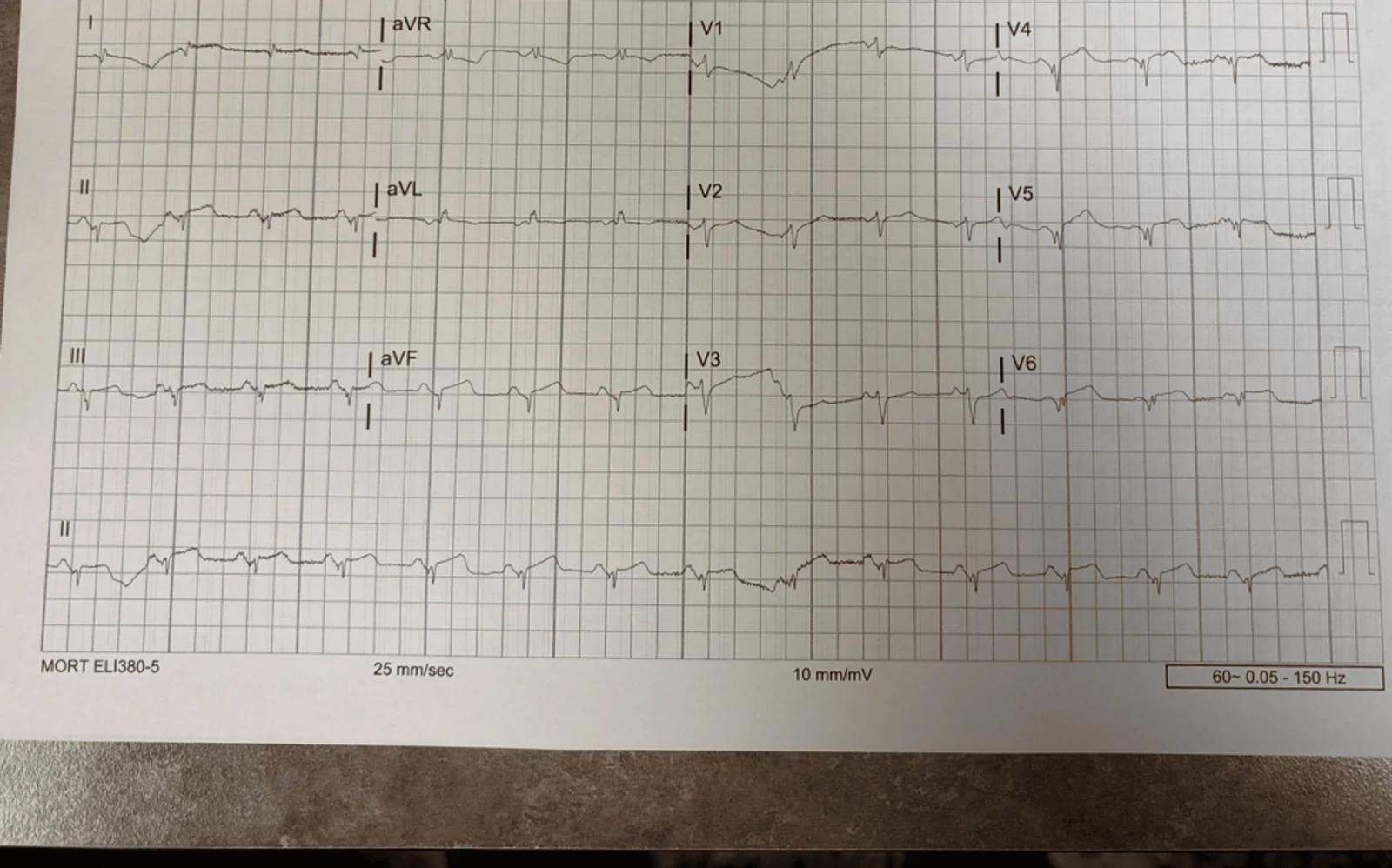
Initial EKG showing ST elevation with pathologic Q waves in inferior leads and anterolateral leads

**Figure 2:**
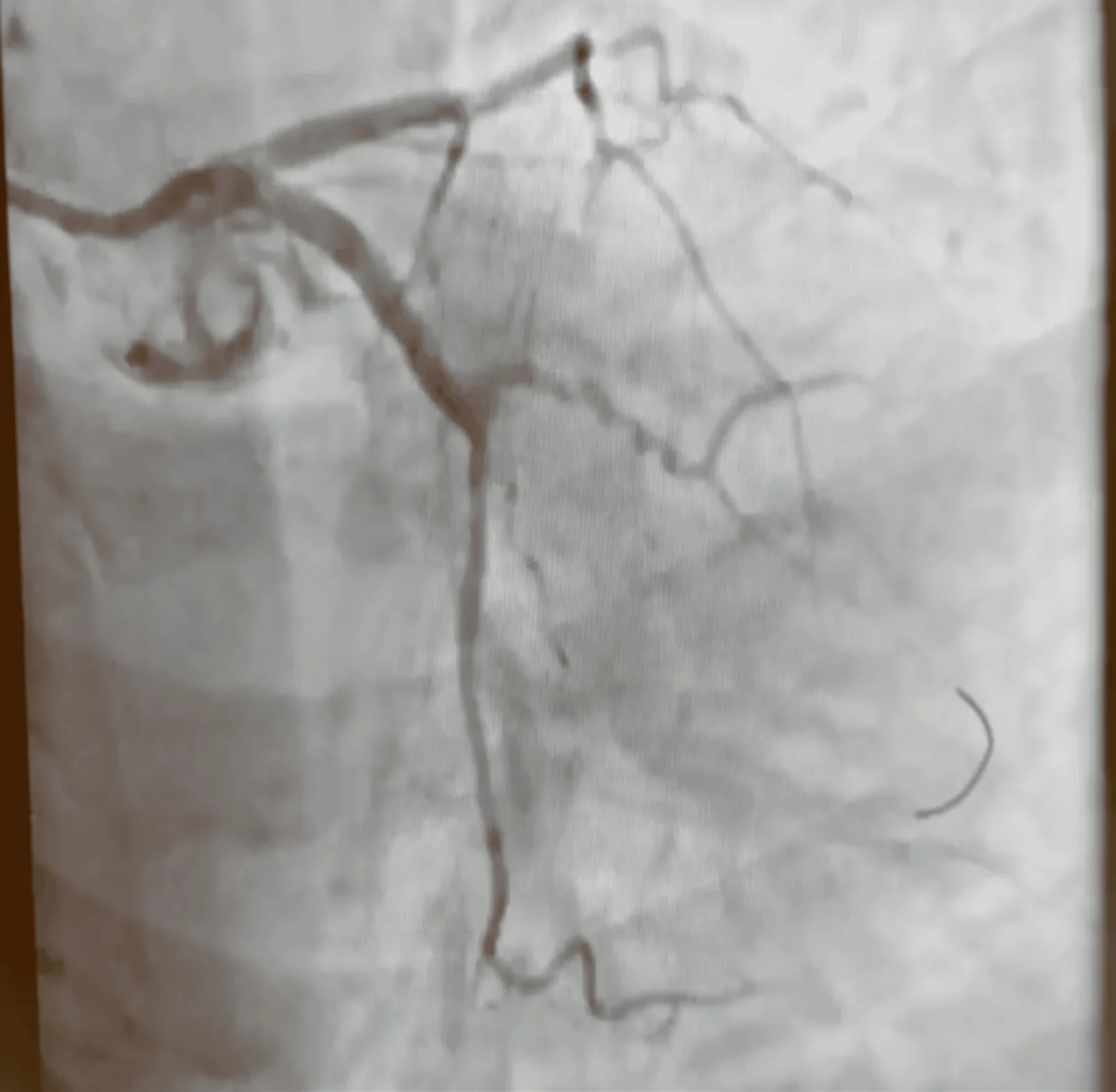
Coronary angiogram showing an embolus with two truncations in distal obtuse marginal 1

**Figure 3:**
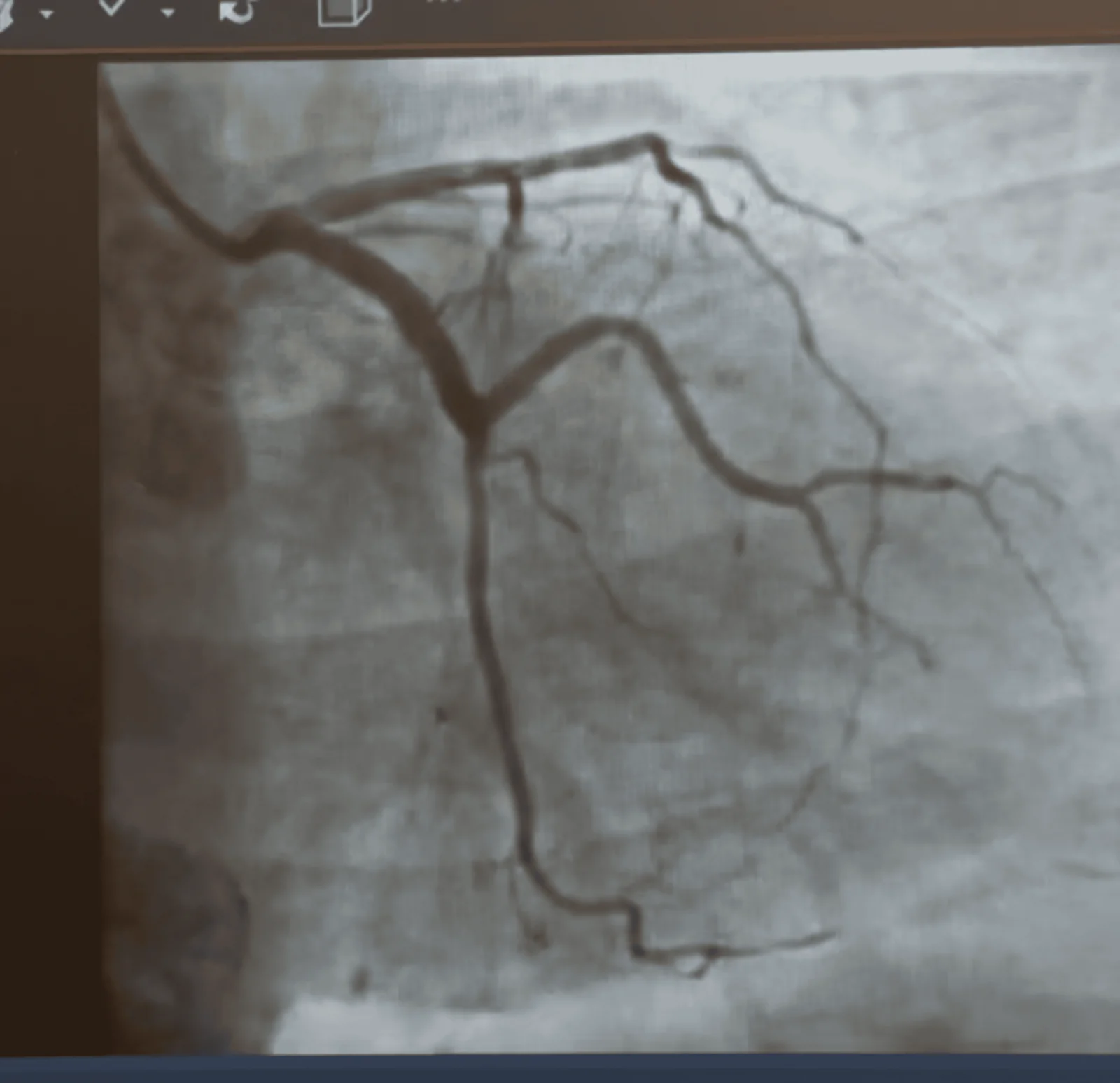
Repeat coronary angiogram demonstrating resolution of the obstruction in the distal obtuse marginal 1 branch

**Figure 4:**
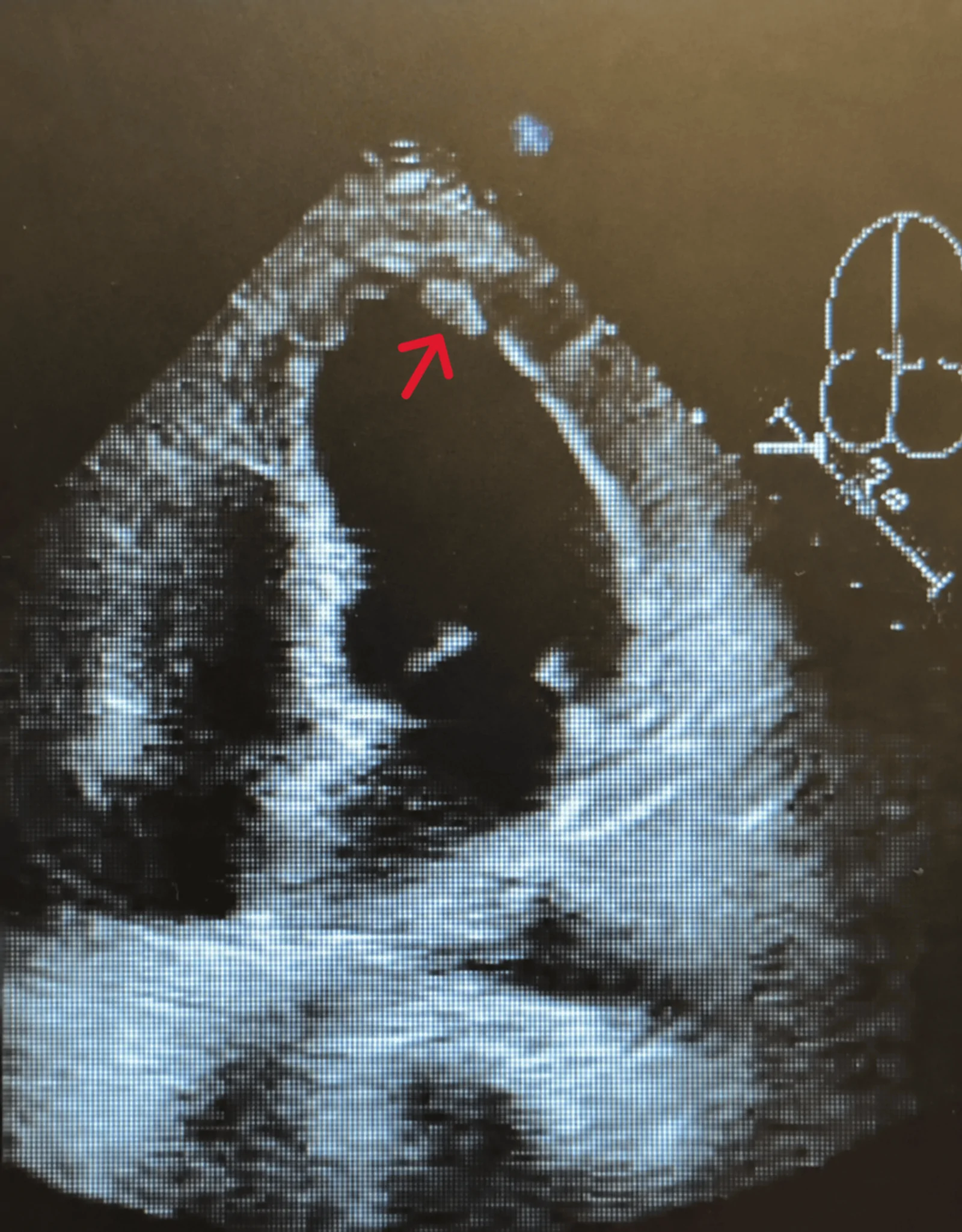
Transthoracic echocardiogram showing apical ballooning with ejection fraction (EF) of 15-20%, consistent with Takotsubo cardiomyopathy and presence of LV mural thrombus (red arrow)

A 74-year-old woman with a history of chronic obstructive pulmonary disease (COPD), tobacco use disorder, dyslipidemia, generalized anxiety disorder, history of unprovoked deep vein thrombosis (DVT), and pulmonary embolism (PE) on apixaban presented to the ER with acute-onset and persistent chest pain and shortness of breath for two days. The patient underwent laser cataract surgery two days prior, for which apixaban was held for three days. The patient reported having significant generalized anxiety disorder and perceived any medical procedure as a stress-producing event. Upon arrival to the ED, EKG showed ST elevation and pathologic Q waves in inferior and anterolateral leads, consistent with ST-elevation myocardial infarction (Figure 1). Initial troponin was 5,827 ng/L (normal was < 14 ng/L), and B-type natriuretic peptide (BNP) was 10,861 pg/mL (normal was <100 pg/mL). The patient was taken urgently to the catheterization laboratory. Cardiac catheterization revealed an embolus with two truncations in the distal obtuse marginal 1 (OM1) branch requiring balloon angioplasty and thrombectomy without evidence of atherosclerotic obstructive coronary artery disease (Figure 2). The patient was given a loading dose of tirofiban and clopidogrel, along with one dose of IV furosemide intraoperatively. After thrombectomy, repeat coronary angiogram demonstrated resolution of obstruction in distal OM1 branch (Figure 3). The transthoracic echocardiogram (TTE) showed a classic TTC picture with basal hyperdynamic and ballooning out of the apex and anterior wall with presence of LV mural thrombus (Figure 4). PE was ruled out by computed tomography pulmonary angiogram (CTA/PE), and home dose apixaban was re-initiated within 24 hours after the procedure.

**Figure 5:**
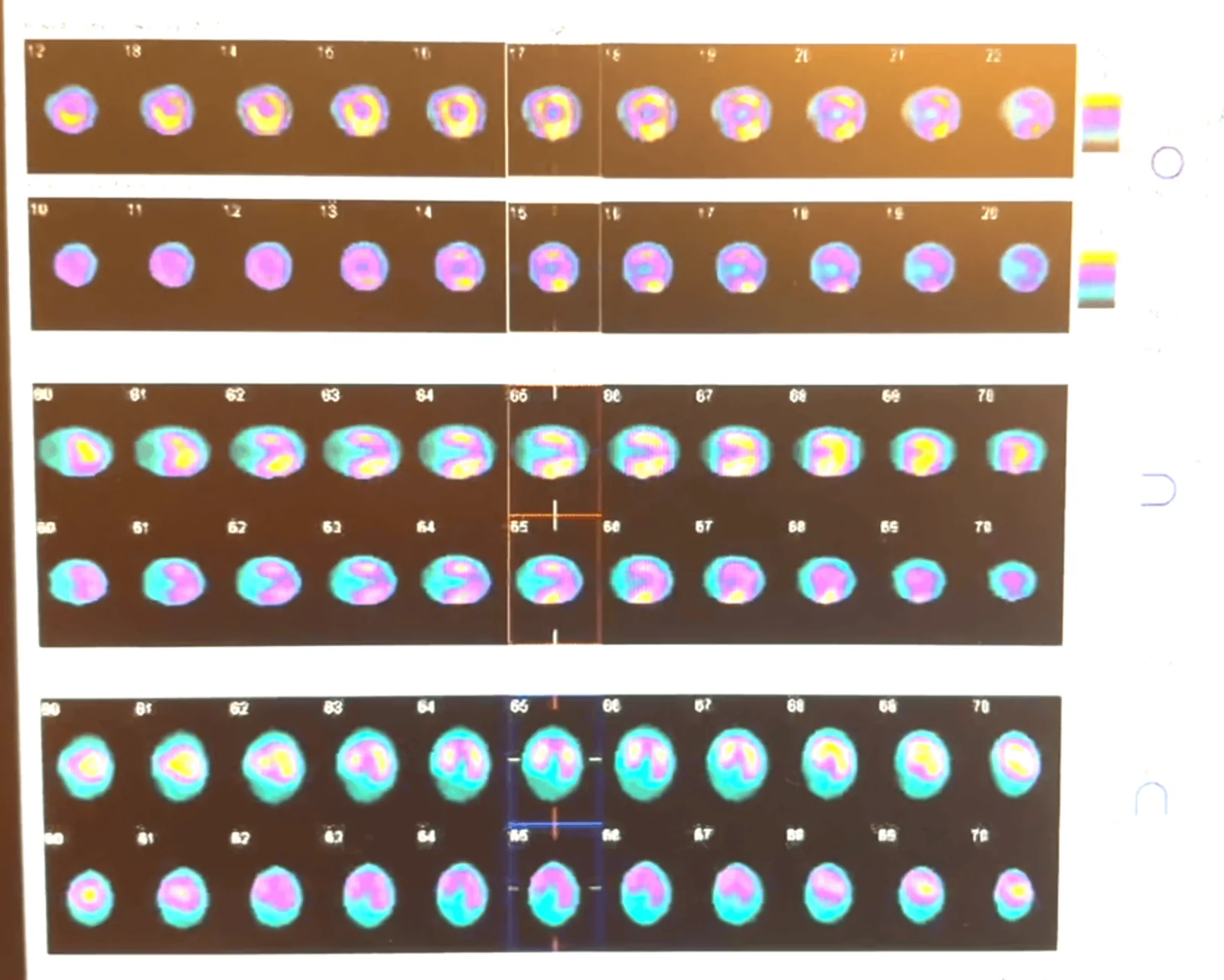
Nuclear stress test two years ago showing a two-grade reversible ischemia in the apical anterior wall and a one-grade reversible ischemia in the anteroseptal wall with a normal stress ECG

TTE 1.5 years prior showed a normal ejection fraction (EF) of 55-60% without wall motion abnormality or dysfunction. Nuclear stress test at the same time showed a two-grade reversible ischemia in the apical anterior wall and a one-grade reversible ischemia in the anteroseptal wall with a normal stress ECG (Figure 5).

The patient was diagnosed with coronary embolism to the OM1 secondary to Takotsubo cardiomyopathy with LV mural thrombus and was treated with dual antiplatelet therapy, aspirin 81 mg daily and clopidogrel 75 mg daily, apixaban 5 mg twice a day, guideline-directed medical therapy (GDMT) including valsartan/sacubitril 24/26 mg twice a day and carvedilol 6.25 mg twice a day, and a wearable defibrillator vest upon discharge.

## Discussion

The development of acute shortness of breath and chest pain indicated a cardiopulmonary etiology until proven otherwise. However, both TTC and coronary embolism can present with these symptoms, and it is difficult to distinguish if the symptoms were caused by one disease or both; other differentials include PE and aortic dissection. Similarly, both TTC and coronary embolism cause ST-elevation, and thus it is difficult to differentiate the etiology. Given the temporal relationship of the stressful event, clinical presentation, evidence of myocardial injury, imaging findings, and lack of alternative explanation, the patient meets all diagnostic criteria for TTC (Figure 4). Her coronary angiogram showed absence of atherosclerotic obstructive coronary artery disease with the presence of a coronary embolus (Figure 2). The ACC/AHA recommends standard heart failure therapy with guideline-directed medical therapy. With appropriate treatment, most patients recover LV function within one to four weeks, but a recurrence rate of 1.8% per year and a major adverse cardiac and cerebrovascular event (MACCE) rate of 9.9% per year have been reported [4].

Currently, coronary embolism is only reported in very few cases [5]. Unlike TTC, there are no standardized diagnostic criteria for coronary embolism. The diagnosis is mainly based on a combination of angiographic findings, exclusion of atherosclerotic disease, and identification of an embolic source. In this case, the development of TTC greatly reduced the blood flow going through the left ventricle (stasis). In the presence of hypercoagulability given the patient's medical history, two of Virchow's triad contributed to the development of the LV mural thrombus. What was atypical in this case was the development of coronary embolism before the return of ventricular mobility. In a typical situation, the recovery of LV function from TTC increases the turbulence of blood flow in the left ventricle, which increases the risk of embolus dislodgement, making this case more rare. The exact mechanism leading to coronary embolism remains unclear. Management of coronary embolism depends on embolus size and location. The SCAI recommends prioritization of antithrombotic or anticoagulation with identification of the underlying source for small and distal emboli. Proximal and large emboli require PCI with angioplasty or thrombus aspiration. Comprehensive evaluation for the source of emboli is necessary to prevent recurrence, and long-term anticoagulation is needed when atrial fibrillation or other thromboembolic risk factors are present [6].

It has been well identified that apical ballooning pattern, reduced left ventricular ejection fraction, elevated troponin, and inflammatory markers are the independent predictors of left ventricular thrombus in Takotsubo cardiomyopathy [7]. The general population of patients with Takotsubo cardiomyopathy has 2-3.3% of acute LV thrombus formation [7]. One third of these patients develop embolic complications, most commonly cerebral or systemic embolism, with coronary embolism being exceedingly rare [8]. The overall rate of any embolic event in Takotsubo patients is therefore less than 1% of all Takotsubo cases within the first year. This patient has multiple other thromboembolic risk factors, including active tobacco use, history of unprovoked venous thromboembolism (VTE), holding apixaban for three days, and a newly emerging pulmonary nodule, suggesting a possible malignant process. Theoretically, in patients with a history of unprovoked VTE, interruption of anticoagulation increases the risk of recurrent VTE, but there is no direct evidence that it increases the risk of LV thrombus specifically in Takotsubo cardiomyopathy. Smoking remains one of the most highlighted reasons for an acquired pro-thrombotic state. A meta-analysis of 21 studies showed that ex-smokers have a relative risk of 1.05 (95% CI: 1.01-1.10) for developing DVT [9]. However, there is currently no evidence that smoking increases the risk of developing left ventricular thrombus in patients with Takotsubo cardiomyopathy. 

It is questionable why apixaban was held for three days for a minimally invasive surgery. According to the CHEST guidelines from 2022, for patients receiving apixaban who require an elective surgery or procedure, the recommended interruption interval depends on the procedural bleeding risk. For low-bleed-risk procedures, such as cataract surgery, apixaban should be stopped for one day before the procedure instead of three days [10]. A PRISMA-based systematic review reported a rebound phenomenon with cessation of direct oral anticoagulation therapy (DOAC) [11]. They screened 1500 medical records (case reports, case series and original records) in patients on DOAC with VTE, atrial fibrillation and other indications. Thrombotic events (myocardial infarction, stroke, systemic embolism and venous thromboembolism) occurred shortly after DOAC cessation. This study highlights the importance of avoiding abrupt or unnecessary discontinuation of DOAC unless clinically indicated. Thus, it is likely multifactorial that the patient developed Takotsubo cardiomyopathy complicated by coronary embolism given her postmenopausal age, recent stressful event, lack of anticoagulation, and being in a hypercoagulable state.

## Conclusions

This case highlights a rare, complex, multifactorial coronary embolism from Takotsubo cardiomyopathy. A joint effort is required from a multidisciplinary team to prevent patients with extremely high risk from developing potentially lethal VTEs. Currently, there is no guideline recommendation regarding stress assessment tools. Development of objective assessment tools may prevent high-risk patients from such medical complications. Further studies need to be done to evaluate the incidence of rebound phenomena to calculate a safe window period for stopping and restarting DOACs in patients with hypercoagulable states.
